# Hepatocellular Carcinoma in Children and Adolescents: Clinical Characteristics and Treatment

**DOI:** 10.1007/s11605-017-3420-3

**Published:** 2017-04-10

**Authors:** Juncheng Wang, Yize Mao, Yongcheng Liu, Zhenxin Chen, Minshan Chen, Xiangming Lao, Shengping Li

**Affiliations:** 1 Department of Hepatobiliary Oncology, State Key Laboratory of Oncology in South China; Collaborative Innovation Center for Cancer Medicine, Sun Yat-sen University Cancer Center, No. 651 Dongfeng Road East, Guangzhou, 510060 China; 2 Department of Surgical Oncology, Sir Run Run Shaw Hospital, Zhejiang University School of Medicine, Hangzhou, 310020 China

**Keywords:** Hepatocellular carcinoma, Children and adolescents, Prognosis, Resection, Transarterial chemoembolization

## Abstract

**Background:**

Hepatocellular carcinoma (HCC) occurs rarely in children and adolescents (C&A), and its clinical characteristics, prognostic factors, and treatment were rarely explored.

**Methods:**

This retrospective study focused on 65 HCC patients aged ≤20 years from August 1994 to August 2012. Cox regression models and Kaplan–Meier curves were used to investigate prognostic factors and compare overall survival (OS), respectively.

**Results:**

We found 61.5% of patients to have multiple tumors, 30.8% to have portal vein tumor thrombus, and 16.9% to have distant metastasis. Diameter of tumors was 10.2 ± 4.1 cm. OS at 5 years was 15.8%. Multivariate analyses showed initial treatment (*P* < 0.001) to be a predictor for OS. For moderate-stage HCC, the median OS of patients who underwent resection was longer than that of patients who underwent transarterial chemoembolization (TACE) or supportive treatment (ST) (*P* < 0.001). For advanced-stage HCC, the median OS of patients who underwent TACE was longer than that of patients who underwent ST (*P* = 0.045).

**Conclusions:**

HCC in C&A tends to be more advanced than that in adults, and resection remains the mainstay of treatment for those patients. Moreover, compared with ST, TACE may benefit C&A with moderate- and advanced-stage HCC, which needs further study.

**Electronic supplementary material:**

The online version of this article (doi:10.1007/s11605-017-3420-3) contains supplementary material, which is available to authorized users.

## Introduction

Liver cancer is the second most common cause of cancer-related deaths worldwide, and there are approximately 850,000 new cases per year worldwide.^1^,^2^ Hepatocellular carcinoma (HCC) is the most common type of hepatic malignancy, but only 0.5–1% of those aged ≤20 years suffer from HCC.^3^

Infection by the hepatitis B virus (HBV) is endemic in China. The incidence of HCC in children and adolescents (C&A) is higher than that for hepatoblastoma, a pattern that is different from that reported in Western countries.^4^,^5^ Unlike HCC in adults, which commonly arises in the setting of prolonged chronic hepatitis and cirrhosis,^6^ tumors in C&A have been observed with HBV infection acquired perinatally or due to inherited metabolic disorders.^7^,^8^ Moreover, at the time of diagnosis, HCC in C&A is commonly associated with (i) huge hepatomas, (ii) tumor thrombus in the portal vein, and (iii) distant metastasis.^7^,^9^ Taken together, such reports suggest that HCC in C&A may be different to that observed in adults.^4^,^8^,^10^ Hence, discovering the prognostic factors of HCC in C&A may help to improve the diagnosis and treatment of HCC.

Even though HCC in C&A exhibits a more malignant tendency, treatment guidelines are lacking. Resection is considered to be the mainstay of curative therapy for long-term survival of C&A with HCC.^3^,^11^–^13^ Nevertheless, the efficiency of transarterial chemoembolization (TACE), which is the established treatment for unresectable HCC in adults,^14^–^16^ for C&A with HCC is controversial.^3^ More specifically, although promising, experience with TACE in C&A with HCC is limited, with only sporadic reports in the literature.^3^,^10^ Moreover, most of those studies have suggested that TACE may have minimal effects on survival,^4^,^17^ and only one study showed positive effects in inducing resection of HCC in C&A.^18^ As a result, the role of TACE for HCC in C&A is still unclear.

In the present study, we analyzed (retrospectively) the clinical and pathologic characteristics of HCC in C&A and evaluated prognostic factors that may help predict survival. Moreover, we compared the outcome of HCC in C&A who underwent different types of treatment to provide evidence for future therapy.

## Patients and Methods

### Patients

This retrospective study was approved by the Institutional Review Board of Sun Yat-sen University Cancer Center (Guangzhou, China).

From 15 August 1994 to 15 August 2012, 72 patients aged ≤20 years were diagnosed with HCC by histological examination or at least two coincidental imaging techniques associated with increased alpha-fetoprotein (AFP) level in our Department of Hepatobiliary Oncology. Patients lost to follow-up <1 month after diagnosis (*n* = 6) or those who did not have sufficient clinical data (*n* = 1) were excluded, thereby leaving 65 patients to form the study cohort.

### Follow-Up

The follow-up program for patients was every 2–3 months for the first postoperative year and 3–6 months thereafter. “Overall survival” (OS) was defined as the interval (in months) from the date of diagnosis to the date of death. Follow-up ended on 1 August 2016.

### Collection of Clinical Data

All clinicopathologic data (age, sex, tumor number, tumor size, distant metastasis, portal vein tumor thrombus, TNM stage) were retrieved from the medical records of Sun Yat-sen University Cancer Center. Laboratory data (HBV infection; levels of AFP, alanine aminotransferase [ALT], aspartate aminotransferase [AST], albumin [ALB], total bilirubin [TBIL]) before surgery were obtained. Tumor stage was determined according to the 7th TNM staging system established by the Union for International Cancer Control and the American Joint Committee on Cancer.^19^

### Statistical Analyses

For continuous variables, data were expressed as mean ± standard deviation and were compared using Student’s *t* test (two-sided). The Cox proportional hazards model was used for univariate and multivariate analyses. Clinical endpoints were calculated using the Kaplan–Meier method with the log-rank test. Analyses were carried out using the SPSS software program (version 20.0; IBM Corporation, Armonk, NY, USA). *P* <0.05 (two-tailed test) was considered significant.

## Results

### Survival and Characteristics of Patients

Median follow-up was 9.1 (range, 1.2–110.2) months. Survival at 1, 3, and 5 years was 50.1, 27.5, and 15.8%, respectively.

Sixty-five patients (52 males, 13 females) with a median age of 16.8 (range, 8–20) years were evaluated (Table 1). Also, 81.5% (53/65) of patients had HBV infection, 61.5% patients had multiple tumors, 16.9% had distant metastasis, and 30.8% had portal vein tumor thrombus. Diameter of tumors was 10.2 ± 4.1 cm, and 90.8% of patients were AFP-positive.

**Table 1:** Baseline characteristics of C&A patients with hepatocellular carcinoma (HCC)

Variable	
Age	17 (8–20)
Sex	
Female	13 (20%)
Male	52 (80%)
HBV infection	
No	12 (18.5%)
Yes	53 (81.5%)
ALT (U/L)	64.5 ± 81.6
AST (U/L)	94.3 ± 130.6
ALB (g/L)	42.9 ± 5.2
TBIL (μmol/L)	22.4 ± 25.4
Tumor diameter (cm)	10.2 ± 4.1
AFP (ng/mL)	
≤25	6 (9.2%)
>25	59 (90.8%)
Tumor number	
Solitary	25 (38.5%)
Multiple	40 (61.5%)
Distant metastasis	
No	54 (83.1%)
Yes	11 (16.9%)
Portal vein tumor thrombus	
No	45 (69.2%)
Yes	20 (30.8%)
TNM stage	
I	9 (13.8%)
II	8 (12.3%)
IIIA	22 (33.8%)
IIIB	14 (21.5%)
IVA	1 (1.5%)
IVB	11 (16.9%)
Initial treatment	
ST	16 (24.6%)
TACE	23 (35.4%)
Resection	26 (40%)

*HBV* hepatitis B virus, *ALT* alanine aminotransferase, *AST* aspartate transaminase, *ALB* albumin, *TBIL* total bilirubin, *AFP* alpha-fetoprotein, *TNM* tumor-node-metastasis, *ST* supportive treatment, *TACE* transcatheter arterial chemoembolization

### TNM Categorization

**Fig. 1:**
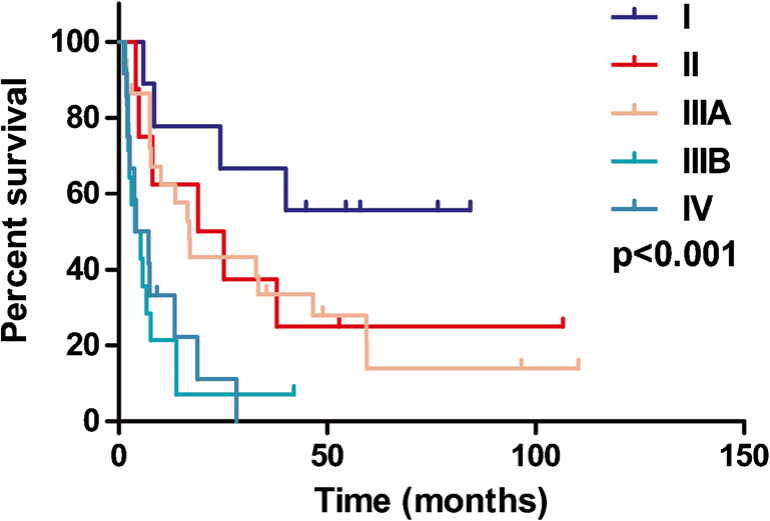
Kaplan–Meier curves for OS of HCC patients with different TNM stages

According to the survival curves, we categorized TNM stage into three subgroups (Fig. 1): stage I was “early,” stages II/IIIA were “moderate,” and stages IIIB/IV were “advanced.”

### Univariate and Multivariate Analyses for OS in HCC Patients

**Fig. 2:**
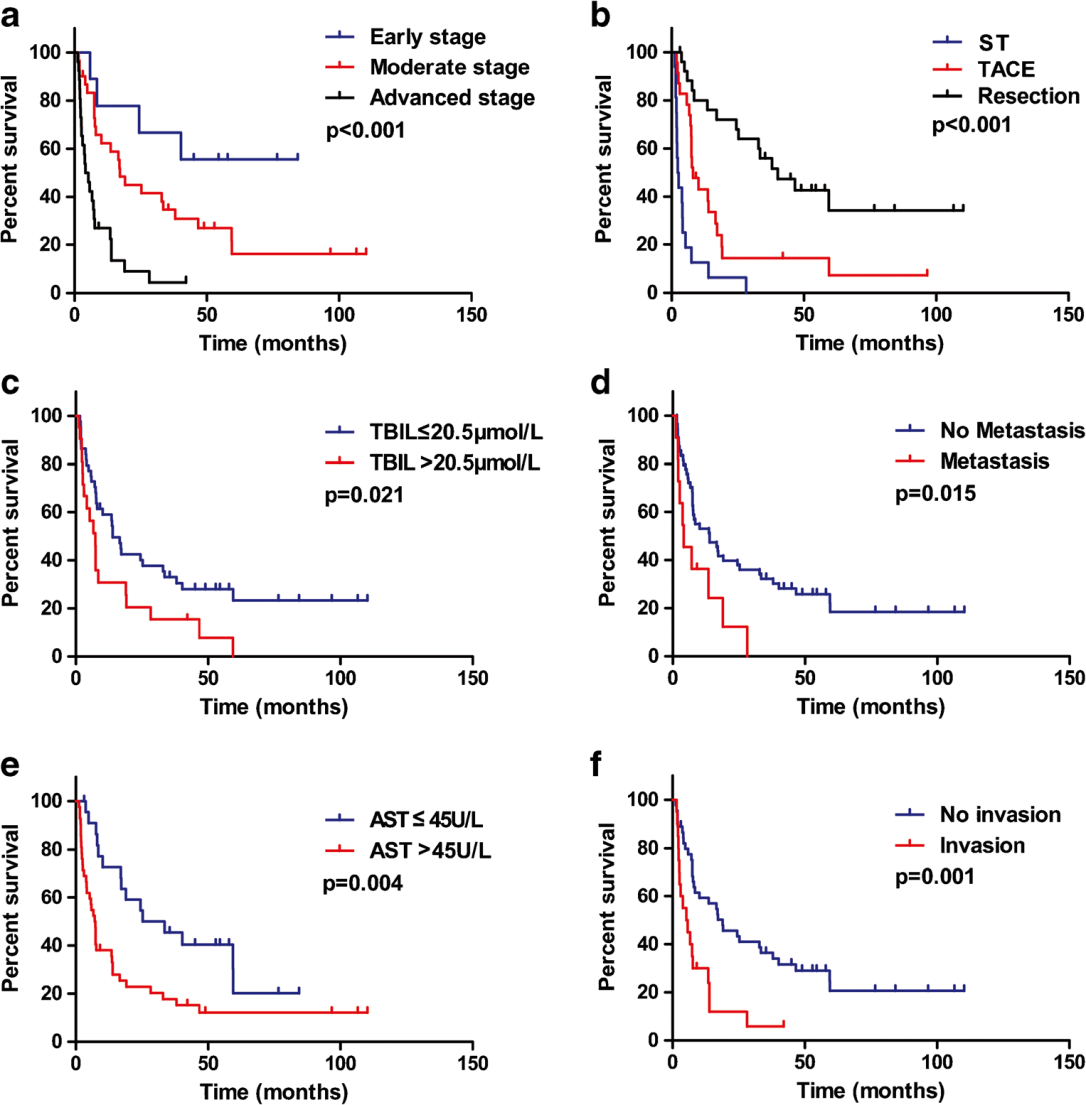
Univariate analyses of prognostic factors using Kaplan–Meier survival curves. **a** Early, moderate, or advanced stage. **b** ST, TACE, or resection. **c** TBIL ≤20.5 or >20.5 μmol/L. **d** Metastasis: no or yes. **e** AST ≤45 or >45 U/L. **f** Portal vein tumor thrombus: no or yes

Univariate and multivariate analyses were undertaken to explore the prognostic factors of HCC patients (Table 2, Fig. 2). Results of the Cox regression hazards model for predictors of OS are shown in Table 2.

**Table 2:** Univariate and multivariate analyses of overall survival (OS)

Characteristic	Univariate		Multivariate	
	HR (95% CI)	*P*	HR (95% CI)	*P*
Sex				
Female	1 (reference)	0.274		
Male	1.529 (0.714–3.274)			
HBV infection				
No	1 (reference)	0.306		
Yes	1.461 (0.707–3.021)			
Tumor number				
Solitary	1 (reference)	0.882		
Multiple	1.068 (0.604–1.886)			
TNM stage				
Early	1 (reference)	<0.001		0.072
Moderate	2.420 (0.835–7.014)			
Advanced	7.479 (2.503–22.342)			
Initial treatment				
ST	1 (reference)	<0.001	1 (reference)	<0.001
TACE	0.298 (0.150–0.592)		0.298 (0.150–0.592)	
Resection	0.105 (0.048–0.226)		0.105 (0.048–0.226)	
Distant metastasis				
No	1 (reference)	0.018		0.448
Yes	2.360 (1.156–4.815)			
Portal vein tumor thrombus				
No	1 (reference)	0.001		0.184
Yes	2.725 (1.484–5.004)			
Tumor diameter (cm)				
≤5	1 (reference)	0.215		
>5	1.912 (0.687–5.323)			
TBIL (μmol/L)				
≤20.5	1 (reference)	0.023		0.944
>20.5	1.947 (1.094–3.465)			
AST (U/L)				
≤45	1 (reference)	0.006		0.786
>45	2.365 (1.287–4.346)			
ALT (U/L)				
≤40	1 (reference)	0.059		
>40	1.702 (0.979–2.959)			
ALB (g/L)				
≤35	1 (reference)	0.457		
>35	0.676 (0.242–1.893)			
AFP (g/L)				
≤25	1 (reference)	0.606		
>25	1.309 (0.47–3.649)			

*HBV* hepatitis B virus, *TNM* tumor-node-metastasis, *ST* supportive treatment, *TACE* transcatheter arterial chemoembolization, *TBIL* total bilirubin, *AST* aspartate transaminase, *ALT* alanine aminotransferase, *ALB* albumin, *AFP* alpha-fetoprotein

Univariate analyses showed that TNM stage (moderate stage vs. early stage: HR 2.420, 95% confidence interval [CI] 0.835–7.014; advanced stage vs. early stage: HR 7.479, 95% CI 2.503–22.342, *P* < 0.001), initial treatment (TACE vs. supportive treatment [ST]: HR 0.298, 95% CI 0.150–0.592; resection vs. ST: HR 0.105, 95% CI 0.048–0.226, *P* < 0.001), metastasis (HR 2.360, 95% CI 1.156–4.815, *P* = 0.015), portal vein tumor thrombus (HR 2.725, 95% CI 1.484–5.004, *P* = 0.001), TBIL level (HR 1.947, 95% CI 1.094–3.465, *P* = 0.021), and AST level (HR 2.365, 95% CI 1.287–4.346, *P* = 0.004) were associated with OS. Multivariate analyses showed only initial treatment allocation (TACE vs. ST: HR 0.298, 95% CI 0.150–0.592; resection vs. ST: HR 0.105, 95% CI 0.048–0.226, *P* = 0.001) to be a predictor of OS.

**Fig. 3:**
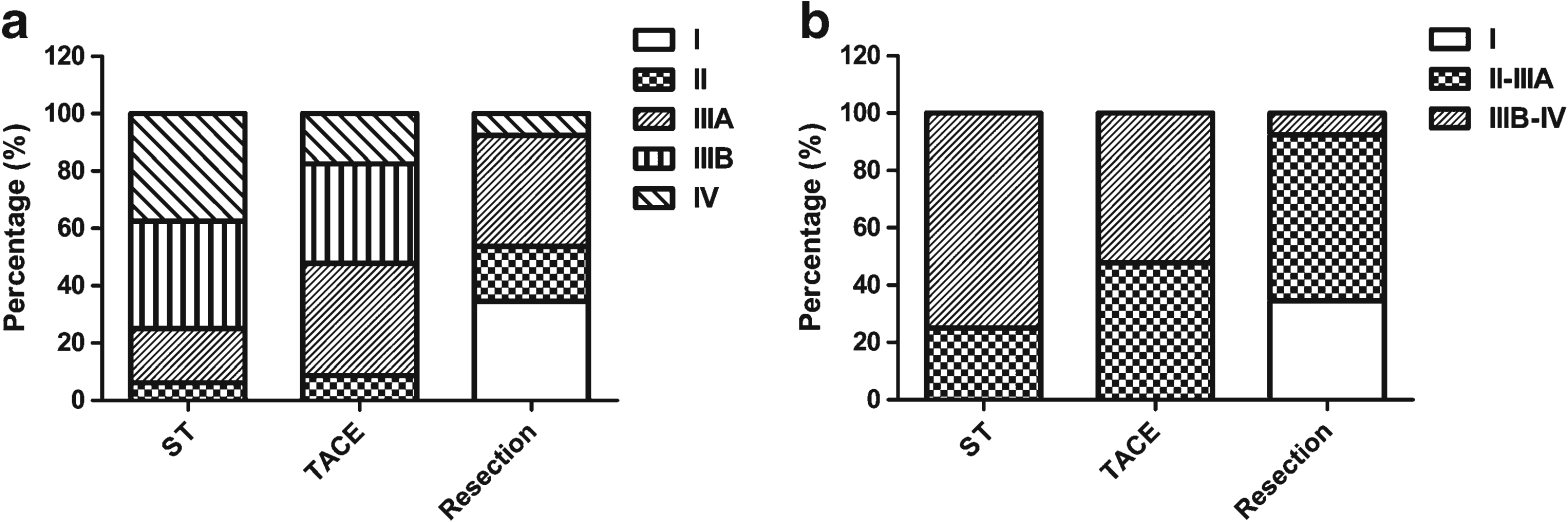
**a** Distribution of TNM stage for HCC in C&A was different for different treatment types (*χ* ^2^ < 0.001). **b** Distribution of combined TNM stage for HCC in C&A was different for different treatments (*χ* ^2^ < 0.001)

To further investigate the relationship between the option of initial treatment and OS, we found that more patients with early or moderate stage received surgery, while a high proportion of the patients with moderate or advanced stage received TACE or ST (*P* < 0.001, Fig. 3). These indicated that the uneven distribution of TNM stage may influence the OS of patients undergoing different types of initial treatment.

### Survival

**Fig. 4:**
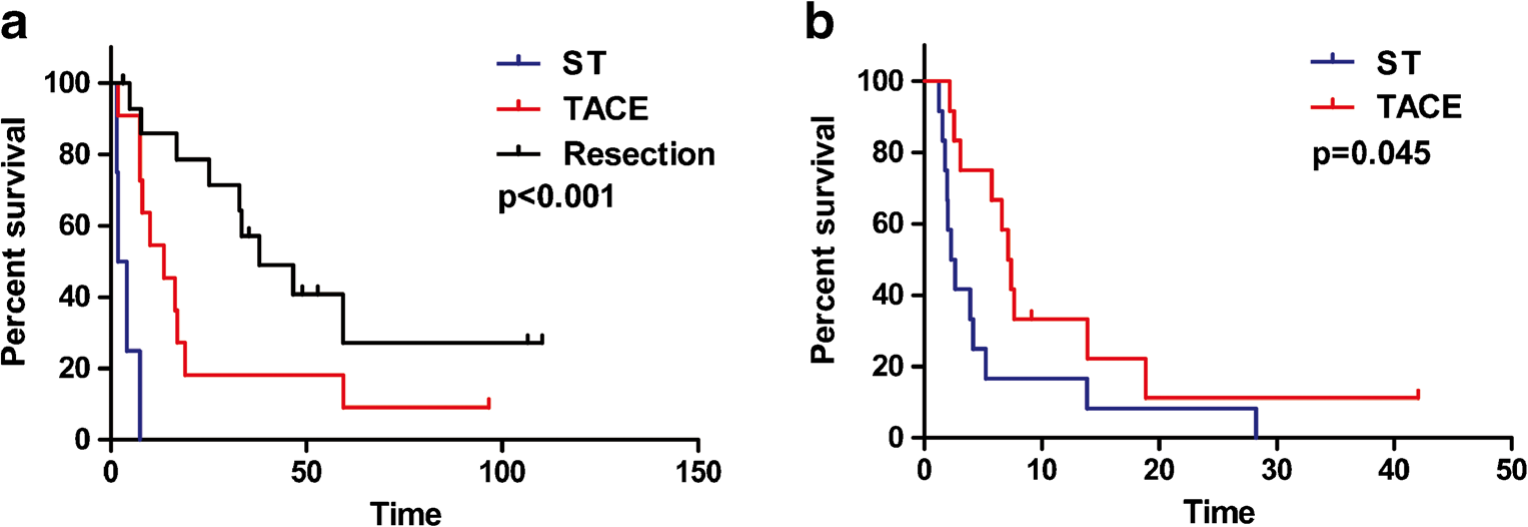
**a** Kaplan–Meier curves for OS of HCC patients at the moderate stage. Median OS of patients who underwent resection was longer than that of patients who underwent TACE or ST (38.0, 13.6, and 1.8 months, respectively, *P* < 0.001). **b** Kaplan–Meier curves for OS of HCC patients with advanced HCC. Median OS of patients who underwent TACE was longer than that of patients who underwent ST (7.1 vs. 2.3 months, *P* = 0.045). The *black curve*, *red curve*, and *blue curve* represent patients who underwent resection, TACE, and ST, respectively

To exclude the influence of the different distribution of TNM stage on initial treatment subgroups, we compared the OS of patients at moderate or advanced stages that underwent different types of treatment. Kaplan–Meier analyses showed that, at the moderate stage, the median OS of patients who underwent resection was longer than that of patients who underwent other types of treatment (resection vs. TACE vs. ST: 38.0 vs. 13.6 vs. 1.8 months, *P* < 0.001, Fig. 4a). At the advanced stage, the OS of patients who underwent TACE was longer than that of patients who underwent ST (TACE vs. ST: 7.1 vs. 2.3 months, *P* = 0.045, Fig. 4b).

## Discussion

The present study demonstrated that C&A with HCC tend to have advanced disease, and initial treatment allocation was found to be an independent prognostic factor. Resection could achieve long-term survival at the moderate stage compared with other types of treatment. Moreover, compared with ST, TACE could have beneficial effects on OS for the advanced stage of HCC.

Several differences with respect to clinical characteristics and outcomes between HCC in C&A and that in adults have been reported.^4^,^20^ Our results identified HBV infection to be present in 81.5% of C&A with HCC, which is similar to the prevalence of adult HCC in our hospital.^21^,^22^ In accordance with other studies, we found that HCC was more common in males than in females (4:1).^3^,^5^ AFP is a useful diagnostic marker for HCC, and roughly 50–70% of adults with HCC have increased levels of AFP,^3^,^23^ compared with >90% in C&A in our study. Moreover, some studies have suggested higher serum levels of AFP to be correlated with worse outcome in HCC.^24^–^26^ We showed that 81.5% of C&A with HCC had AFP >400 ng/mL, which suggests that C&A tend to present with more advanced disease compared with adults. However, we did not find a significant difference in OS between the two groups (AFP >400 vs. ≤400 ng/mL; median OS 7.7 vs. 28.2 months, *P* = 0.075), which might be due to the small sample size. Recent studies have shown serum levels of AFP to be associated with tumor diameter,^27^–^29^ as did our data (*r* = 0.261, *P* = 0.035) (Fig. S1), with 58 (89.2%) patients having a tumor diameter >5 cm and 34 (52.3%) patients having a tumor diameter ≥10 cm. Moreover, multiple nodules were identified in 40 (61.5%) C&A patients. Recent studies have shown the prevalence of distant metastasis and tumor thrombus in the portal vein to be higher in C&A than that in adults, which was correlated with worse outcomes.^1^,^3^^,^ 4 We also showed that 11 (16.9%) patients had distant metastasis and 20 (30.8%) patients had tumor thrombus in the portal vein upon first hospital admission. Moreover, our findings showed that HCC in C&A carried a dismal 5-year OS of 15.8%, which is lower than that observed in adults.^8^,^30^ These findings suggest that HCC in C&A tends to present with more advanced disease than that in adults.

Even though HCC in C&A tends to be more malignant, treatment guidelines on HCC management are lacking.^3^,^13^ Using univariate and multivariate analyses, we identified initial treatment allocation to be an independent prognostic factor. Different types of initial treatment affected the prognosis of HCC in C&A markedly, with the median survival for resection, TACE, and ST being 40.1, 8.0, and 2.3 months, respectively (*P* < 0.001, Fig. 2b). If stratified by TNM stage, moderate-stage patients who underwent resection had better outcome than those who underwent TACE or those who did have ST (*P* < 0.001, Fig. 4a). For patients with advanced disease, those who underwent TACE had better outcome than those who did have ST (*P* < 0.05, Fig. 4b). TACE has been shown to improve survival for unresectable HCC in adults,^31^,^32^ but its role in C&A with HCC is not known.^3^,^17^ Thus, our study showed that compared with ST, TACE might have an effect on unresectable HCC in C&A. However, it should be noted that the difference in OS between ST and TACE might be based on differences in the clinical condition of patients due to the retrospective nature of the study, and prospective studies are needed to confirm those findings.

In the current retrospective study, none of the patients underwent liver transplantation. Some recent studies showed that liver transplantation might be an effective treatment option although the literature is sparse. The studies of Pham et al. and Malek et al. suggested that liver transplantation for HCC in C&A could result in excellent long-term survival, even though the lesions were well outside the Milan and UCSF criteria.^33^,^34^ However, more studies are needed to verify transplantation as a therapeutic intervention for HCC in C&A. Chemotherapy has little effect in improving survival.^3^,^35^ Preliminary results from the Pediatric Oncology Group (POG) study showed that a cisplatin-based chemotherapy regimen might benefit children with resectable HCC.^20^ However, two subsequent prospective studies from the International Childhood Liver Tumor Study Group (SIOPEL 2 and SIOPEL 3) found that intensification of platinum agents did not result in improved survival.^36^ Moreover, the data about ablative therapies and sorafenib in C&A with HCC is lacking, which needs further study. At present, there is no standard clinical guideline for HCC in C&A, and more prospective studies should be conducted in the future.

In addition, two patients were found to have extrahepatic metastasis after laparotomy (one with a nodule in the gastric omentum and the other with a nodule in the diaphragm). When the tumors were removed successfully, the two patients died at 3.7 and 13.5 months, respectively.

Our study had three main limitations. First, this was a retrospective review of data collected from a single cancer center with a small sample size. Hence, we provided low-grade evidence for future therapy.^37^ Second, apart from one patient, the age of the other patients was 10–20 years old. Therefore, the efficacy of TACE in HCC patients under the age of 10 years must be evaluated further. Third, the efficacy of initial TACE therapy for patients with unresectable HCC might be skewed by subsequent treatments. Nevertheless, this can be deciphered by the better treatment response for initial TACE therapy because the subsequent treatments depended mostly on tumor burden and liver function.^31^

## Conclusions

Compared with HCC in adults, HCC in C&A tends to be more advanced, which results in worse survival in C&A. Initial treatment allocation was found to be an independent prognostic factor for HCC in C&A. Consistent with other studies, our study showed that resection is the recommended choice for early- and moderate-stage HCC when technically feasible. Moreover, we found that, compared with ST, TACE may benefit C&A with moderate- and advanced-stage HCC.

## Electronic Supplementary Material

ESM 1 (DOCX 20 kb)

Fig S1 (GIF 8 kb)

High resolution image (TIFF 909 kb)
