# Supplementary material for: Hepatocellular Carcinoma in Children and Adolescents: Clinical Characteristics and Treatment
Source: J Gastrointest Surg. 2017 Apr 10;21(7):1128–35. doi: 10.1007/s11605-017-3420-3 (PMC5486687; doi:10.1007/s11605-017-3420-3)
Supplement: Supplementary file 1 — (DOCX 20 kb) [file 11605_2017_3420_MOESM1_ESM.docx]

supplemental table

| patients | radical therapy^a^ | TNM stage | prognosis^b^ | OS (months) | initial treatment | therapeutic response | subsequent treatment |
| --- | --- | --- | --- | --- | --- | --- | --- |
| 1 | 1 | I | 0 | 54.4 | HR |  |  |
| 2 | 1 | I | 0 | 44.9 | HR |  |  |
| 3 | 1 | I | 0 | 84.3 | HR |  |  |
| 4 | 1 | I | 0 | 76.6 | HR |  | TACE/PI (pulmonary metastasis)/PI (pulmonary metastasis)/PI (pulmonary metastasis)^c^ |
| 5 | 1 | I | 0 | 57.9 | HR |  |  |
| 6 | 1 | I | 1 | 40.1 | HR |  | TACE/HR/TACE/RFA |
| 7 | 1 | I | 1 | 24.3 | HR |  | TACE |
| 8 | 1 | I | 1 | 5.8 | HR |  | TACE |
| 9 | 1 | I | 1 | 8.5 | HR |  | TACE |
| 10 | 1 | II | 0 | 106.5 | HR |  |  |
| 11 | 1 | II | 0 | 52.9 | HR |  |  |
| 12 | 1 | II | 1 | 25.2 | HR |  |  |
| 13 | 1 | II | 1 | 4.8 | HR |  |  |
| 14 | 1 | II | 1 | 38.0 | HR |  | TACE/PMCT |
| 15 | 0 | IIIA | 0 | 110.2 | HR |  | TACE |
| 16 | 1 | IIIA | 0 | 49.0 | HR |  | HR (metastatic tumor of abdominal wall)/Sorafenib/CT |
| 17 | 1 | IIIA | 0 | 3.1 | HR |  | TACE |
| 18 | 1 | IIIA | 0 | 35.4 | HR |  | TACE+RFA^d^ |
| 19 | 1 | IIIA | 1 | 32.9 | HR |  | PEI/TACE/TACE/TACE |
| 20 | 0 | IIIA | 1 | 46.6 | HR |  |  |
| 21 | 1 | IIIA | 1 | 59.4 | HR |  | HR |
| 22 | 1 | IIIA | 1 | 7.7 | HR |  |  |
| 23 | 1 | IIIA | 1 | 16.9 | HR |  | TACE/TACE |
| 24 | 1 | IIIA | 1 | 33.5 | HR |  | TACE/HR/TACE/PEI |
| 25 | 0 | IVB | 1 | 3.7 | HR |  |  |
| 26 | 0 | IVB | 1 | 13.5 | HR |  | TACE |
| 27 | 0 | II | 1 | 19.0 | TACE | SD | TACE/TACE/TACE/PEI/TACE/TACE/TACE/TACE |
| 28 | 0 | II | 1 | 8.0 | TACE | unknown |  |
| 29 | 0 | IIIA | 0 | 96.6 | TACE | PR | HR/PMCT/CIK |
| 30 | 0 | IIIA | 1 | 59.5 | TACE | PR | TACE/TACE+RFA/TACE/HR/TACE/RFA/TACE+RFA/TACE/RFA/TACE/RFA/PMCT/RFA |
| 31 | 0 | IIIA | 1 | 7.4 | TACE | PR | HR/RFA/RFA |
| 32 | 0 | IIIA | 1 | 10.1 | TACE | PR | TACE |
| 33 | 0 | IIIA | 1 | 17.2 | TACE | PR | TACE/TACE/TACE |
| 34 | 0 | IIIA | 1 | 7.4 | TACE | unknown |  |
| 35 | 0 | IIIA | 1 | 16.5 | TACE | SD | TACE |
| 36 | 0 | IIIA | 1 | 1.9 | TACE | unknown |  |
| 37 | 0 | IIIA | 1 | 13.6 | TACE | SD | TACE |
| 38 | 0 | IIIB | 0 | 42.1 | TACE | unknown | LT/CT (pulmonary metastasis) |
| 39 | 0 | IIIB | 1 | 7.6 | TACE | SD | PEI+TACE/PEI |
| 40 | 0 | IIIB | 1 | 5.7 | TACE | PD | TACE |
| 41 | 0 | IIIB | 1 | 13.9 | TACE | unknown |  |
| 42 | 0 | IIIB | 1 | 3.1 | TACE | PD |  |
| 43 | 0 | IIIB | 1 | 2.2 | TACE | unknown |  |
| 44 | 0 | IIIB | 1 | 2.5 | TACE | PD |  |
| 45 | 0 | IIIB | 1 | 6.6 | TACE | unknown |  |
| 46 | 0 | IVA | 1 | 7.4 | TACE | SD | TACE+PEI |
| 47 | 0 | IVB | 1 | 18.8 | TACE | SD | TACE |
| 48 | 0 | IVB | 1 | 7.1 | TACE | PR | PMCT/PEI (metastatic tumor of pelvic cavity) |
| 49 | 0 | IVB | 0 | 9.1 | TACE | unknown |  |
| 50 | 0 | II | 1 | 4.0 | ST |  |  |
| 51 | 0 | IIIA | 1 | 1.8 | ST |  |  |
| 52 | 0 | IIIA | 1 | 1.5 | ST |  |  |
| 53 | 0 | IIIA | 1 | 7.5 | ST |  |  |
| 54 | 0 | IIIB | 1 | 1.8 | ST |  |  |
| 55 | 0 | IIIB | 1 | 13.9 | ST |  |  |
| 56 | 0 | IIIB | 1 | 2.3 | ST |  |  |
| 57 | 0 | IIIB | 1 | 1.5 | ST |  |  |
| 58 | 0 | IIIB | 1 | 3.9 | ST |  |  |
| 59 | 0 | IIIB | 1 | 5.2 | ST |  |  |
| 60 | 0 | IVB | 1 | 4.2 | ST |  |  |
| 61 | 0 | IVB | 1 | 2.0 | ST |  |  |
| 62 | 0 | IVB | 1 | 1.2 | ST |  |  |
| 63 | 0 | IVB | 1 | 28.2 | ST |  |  |
| 64 | 0 | IVB | 1 | 2.6 | ST |  |  |
| 65 | 0 | IVB | 1 | 2.0 | ST |  |  |

a: 0 NO, 1 YES.

b: 0 Alive, 1 Dead.

c:“/”was used to separate two times of therapy.

d:“+”was used to represent combination therapy of a single treatment.

HR, hepatic resection; TACE, transcatheter arterial chemoembolization; ST, supportive treatment; SD, stable disease; PR, partial response; PD, progressive disease; PI, particle implantation (I^125^); RFA, radiofrequency ablation; PMCT, percutaneous microwave coagulation therapy; CT, chemotherapy; PEI, percutaneous ethanol injection; CIK, cytokine induced killer therapy; LT, liver transplantation.
