# Supplementary figures and images for: Hepatocellular Carcinoma in Children and Adolescents: Clinical Characteristics and Treatment
Source: J Gastrointest Surg. 2017 Apr 10;21(7):1128–35. doi: 10.1007/s11605-017-3420-3 (PMC5486687; doi:10.1007/s11605-017-3420-3)

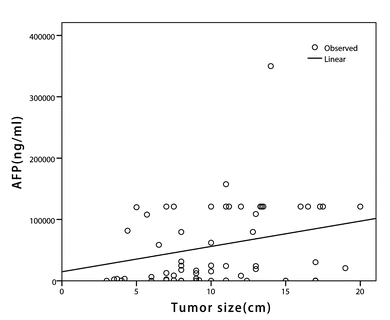

Supplement: Supplementary file 2 — (GIF 8 kb) [file 11605_2017_3420_Fig5_ESM.gif]

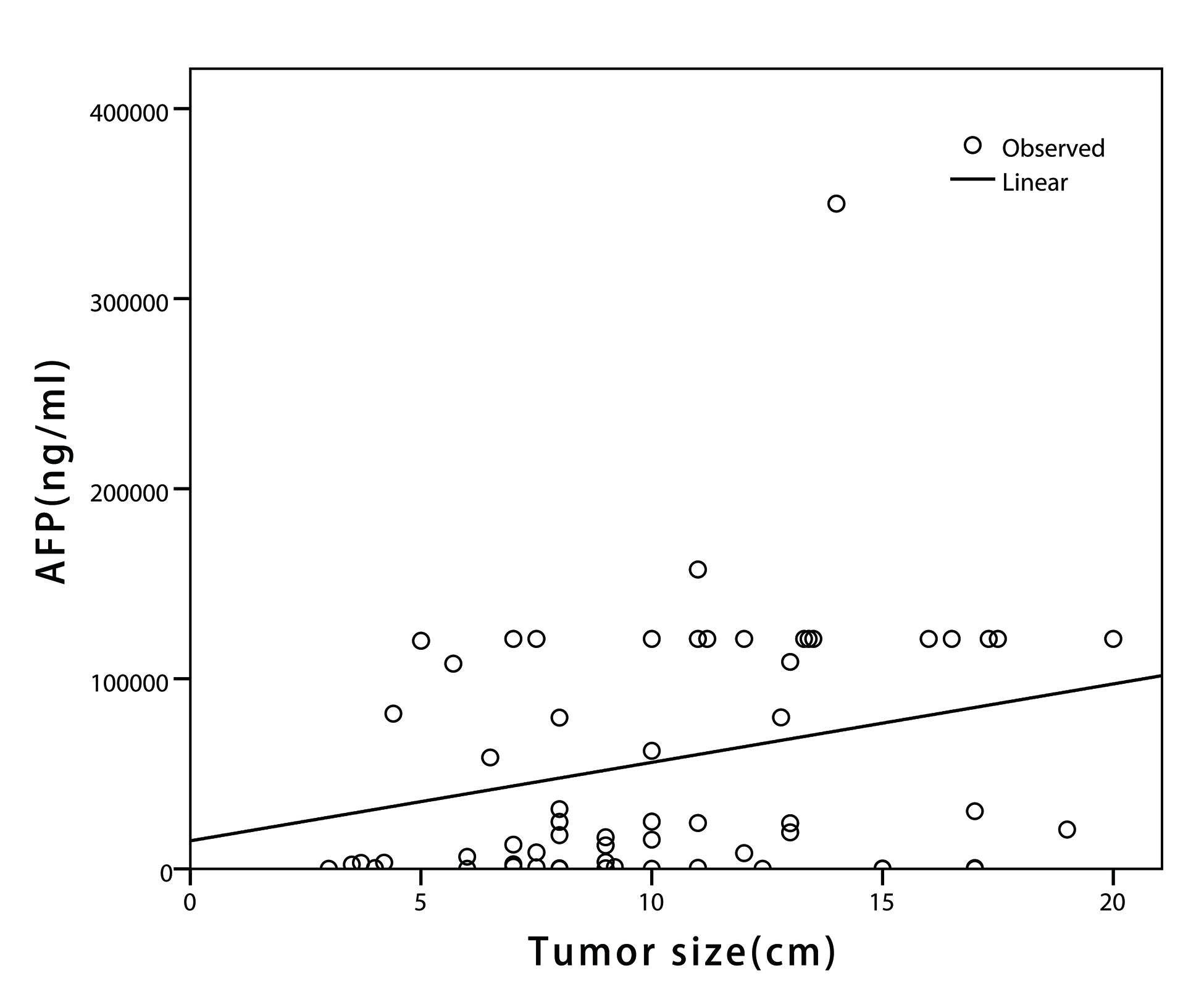

Supplement: Supplementary file 3 — High resolution image (TIFF 909 kb) [file 11605_2017_3420_MOESM2_ESM.tif]
